# A registry of achondroplasia: a 6-year experience from the Czechia and Slovak Republic

**DOI:** 10.1186/s13023-022-02374-x

**Published:** 2022-06-16

**Authors:** Martin Pesl, Hana Verescakova, Linda Skutkova, Jana Strenkova, Pavel Krejci

**Affiliations:** 1 Department of Biology, Faculty of Medicine, Masaryk University, Brno, Czech Republic; 2 International Clinical Research Center, St. Anne University Hospital, Brno, Czech Republic; 3 1st Department of Internal Medicine, Cardioangiology, St. Anne University Hospital and Faculty of Medicine, Masaryk University, Brno, Czech Republic; 4 Department of Pediatrics, University Hospital Brno, Brno, Czech Republic; 5 Institute of Biostatistics and Analyses, Masaryk University, Brno, Czech Republic; 6 Institute of Animal Physiology and Genetics, Czech Academy of Sciences, Brno, Czech Republic

**Keywords:** Skeletal dysplasia, Achondroplasia, FGFR3, Treatment, Registry, ReACH

## Abstract

**Background:**

Achondroplasia (ACH) is one of the most prevalent genetic forms of short-limbed skeletal dysplasia, caused by gain-of-function mutations in the receptor tyrosine kinase FGFR3. In August 2021, the C-type natriuretic peptide (CNP) analog vosoritide was approved for the treatment of ACH. A total of six other inhibitors of FGFR3 signaling are currently undergoing clinical evaluation for ACH. This progress creates an opportunity for children with ACH, who may gain early access to the treatment by entering clinical trials before the closure of their epiphyseal growth plates and cessation of growth. Pathophysiology associated with the ACH, however, demands a long observational period before admission to the interventional trial. Public patient registries can facilitate the process by identification of patients suitable for treatment and collecting the data necessary for the trial entry.

**Results:**

In 2015, we established the prospective ACH registry in the Czechia and the Slovak Republic (http://www.achondroplasia-registry.cz). Patient data is collected through pediatric practitioners and other relevant specialists. After informed consent is given, the data is entered to the online TrialDB system and stored in the Oracle 9i database. The initial cohort included 51 ACH children (average age 8.5 years, range 3 months to 14 years). The frequency of selected neurological, orthopedic, or ORL diagnoses is also recorded. In 2015–2021, a total of 89 measurements of heights, weights, and other parameters were collected. The individual average growth rate was calculated and showed values without exception in the lower decile for the appropriate age. Evidence of paternal age effect was found, with 58.7% of ACH fathers older than the general average paternal age and 43.5% of fathers older by two or more years. One ACH patient had orthopedic limb extension and one patient received growth hormone therapy. Low blood pressure or renal impairment were not found in any patient.

**Conclusion:**

The registry collected the clinical information of 51 pediatric ACH patients during its 6 years of existence, corresponding to ~ 60% of ACH patients living in the Czechia and Slovak Republic. The registry continues to collect ACH patient data with annual frequency to monitor the growth and other parameters in preparation for future therapy.

**Supplementary Information:**

The online version contains supplementary material available at 10.1186/s13023-022-02374-x.

## Background

Achondroplasia (ACH) is one of the most common non-lethal genetic forms of short stature in humans, with an incidence between 1:15,000 to 1:40,000 live births [1–4]. ACH is caused by activating mutations in FGFR3 receptor tyrosine kinase, which transduces the extracellular communication signals mediated by fibroblast growth factors [5]. The ACH mutations are inherited in an autosomal dominant pattern and localize predominantly to the transmembrane and kinase domains of FGFR3. Four other short stature conditions similar to ACH are also caused by FGFR3 mutations, i.e., hypochondroplasia, SADDAN (severe achondroplasia with developmental delay and acanthosis nigricans) and thanatophoric dysplasia type I and type II. These conditions are collectively termed the FGFR3-related skeletal dysplasias [6]. In ACH, the G380R substitution in the transmembrane domain of FGFR3 accounts for 99% of cases [7] and activates FGFR3 signaling through increased spontaneous dimerization and autophosphorylation [8].

Growth plate cartilage chondrocytes are the primary expression site of FGFR3 in mammals [9]. Aberrant activation of FGFR3 alters several signaling pathways necessary for proper chondrocyte proliferation and differentiation such as WNT, cytokine/STAT, BMP, and Hedgehog signaling [10–13]. FGFR3 induces chondrocyte proliferation arrest, degradation of the cartilaginous extracellular matrix, and premature senescence, collectively leading to disruption of growth plate architecture and impaired endochondral ossification [14, 15].

ACH is a non-lethal skeletal dysplasia with an average life expectancy decreased by 10 years [16, 17]. The hallmark clinical features of ACH are disproportionate short stature with an adult height of 112–145 cm, midface hypoplasia due to diminished growth of the base of the skull, rhizomelic limb shortening due to growth plate malfunction, and vertebral pedicle shape alterations [18]. Arising complications include spinal stenosis and hydrocephalus, which stem from changes in the foramen magnum and spine [19–21]. Sleep apnea is attributed either to upper airway obstruction, related to reduced midface hypoplasia [22–24], or myelopathy, related to the cervical spinal cord due to the reduced diameter of the foramen magnum [25–28]. Sudden death from foramen magnum stenosis was reported and has been substantially reduced by neurosurgical interventions [29, 30]. Heart disease‐related mortality between ages 25 and 35 was over ten times higher than the general population and is attributed to obesity [31, 32]. Other complications include acute and chronic otitis media, which can result in hearing impairment and delay in speech acquisition [33].

The first ACH therapeutic, vosoritide (Voxzogo), was approved in August 2021. Vosoritide is based on a C-type natriuretic peptide (CNP), which is a physiological regulator of bone growth [34]. In clinical trials, vosoritide increased average growth velocity gain over natural growth in ACH patients over a period of up to two years [35, 36]. These results are encouraging although long-term vosoritide efficacy (an absolute gain in stature height) is difficult to estimate since the clinical trials only present evidence in ACH children in growth phases between their 5th and 14th year [35]. In addition to vosoritide, a total of six other inhibitors of FGFR3 signaling are being evaluated in ACH clinical trials [37]. These include CNP variants CNP-PEG and ASB20123, as well as conceptually different inhibitors, which target FGFR3 catalytic activity (infigratinib), inhibit FGFR3 downstream signaling (meclizine), or act as extracellular traps for FGFR3 ligands (recifercept, RBM-007).

Patient age is a factor in admission to the trial, as ACH can only be treated prior to epiphyseal growth plate closure. ACH pathophysiology however demands a long observational period before admission to the interventional trial, limiting the chances to enter the trial for many patients. Clinical registries collecting electronic health records can facilitate access to clinical trials by compiling information on comorbidities, family history, social involvement, and psychological aspects [38]. Because ACH is a rare disease it is estimated that only one out of ten pediatricians will take care of any ACH patient during their lifetime career. Thus the standardization of data reporting and analysis in the registries is also necessary [39]. In this article, we report the establishment of the ACH registry in the Czechia and Slovak Republic, and a six-year experience with running the registry.

## Results and discussion

No epidemiological data regarding ACH incidence was available for the Czechia or Slovak Republic before 2015. Based on reported ACH incidence (1/15,000–40,000 live births) [20], we estimate ~ 80 pediatric ACH patients in growth phases under 14 years of age are living in the Czechia and Slovak Republic (combined population 15 million, natality 150,000 children/year). The Registry of Achondroplasia (ReACH; www.achondroplasia-registry.cz) was opened in November 2015. Patient recruitment and reporting were facilitated by educational workshops at the annual meetings of local patient focus groups (ospalecek.cz, paleckovia.sk). The registry gathered a total of 76 patient contacts. The majority of patients had genetically verified ACH, two patients had pseudoachondroplasia, and five patients had hypochondroplasia. Among the 62 pediatric ACH patients were 59 children in growth phase, i.e. aged 14 years or less. A total of 51 patients provided one or more follow-ups (60% of the estimated ACH pediatric population); eight patients were not included in the study due to incomplete data.

All Czech and Slovak ACH patients in the registry carry a G380R substitution in FGFR3, generated by G to A (98% of cases) or G to C (2% of cases) substitution at the *FGFR3* nucleotide 1138. All ACH patients were sporadic cases. This is in contrast to the published evidence reporting 5–20% of familial ACH [40]. This may be due to social stigmatization and institutional trust/distrust in affected families [41]. The median paternal age was acquired from the Czech Statistics Institute only for the region of South Moravia, as this data is not available for the whole Czechia territory. Compared with the median paternal age, the mean difference of ACH paternal age was 2.65 ± 6.4 years (average ± S.D., n = 46). There were 58.7% of ACH fathers older than the general average paternal age. The paternal age effect, defined as a father being two or more years older than the general population [42], was present in 43.5% of fathers (Table 1).

**Table 1:** Paternal age effect in ACH patients

Patient No	Father age	Median age	Difference	PAE
	(yrs)	(yrs)	(yrs)	
1	51	33.9	17.1	Yes
2	50	34.1	15.9	Yes
3	49	34.2	14.8	Yes
4	47	33.9	13.1	Yes
5	47	34.1	12.9	Yes
6	45	33.1	11.9	Yes
7	44	32.4	11.6	Yes
8	44	32.6	11.4	Yes
9	45	33.6	11.4	Yes
10	42	33.1	8.9	Yes
11	39	32.4	6.6	Yes
12	39	33.4	5.6	Yes
13	37	32.4	4.6	Yes
14	38	33.6	4.4	Yes
15	37	32.8	4.2	Yes
16	37	32.8	4.2	Yes
17	38	34.1	3.9	Yes
18	36	32.8	3.2	Yes
19	37	34.1	2.9	Yes
20	36	33.4	2.6	Yes
21	36	34.1	1.9	No
22	35	33.6	1.4	No
23	34	33.1	0.9	No
24	35	34.1	0.9	No
25	35	34.2	0.8	No
26	34	33.4	0.6	No
27	34	33.9	0.1	No
28	33	33.1	− 0.1	No
29	33	33.1	− 0.1	No
30	34	34.1	− 0.1	No
31	34	34.3	− 0.3	No
32	33	33.4	− 0.4	No
33	32	32.6	− 0.6	No
34	32	33.1	− 1.1	No
35	32	33.9	− 1.9	No
36	31	33.1	− 2.1	No
37	31	33.9	− 2.9	No
38	30	33.4	− 3.4	No
39	28	31.7	− 3.7	No
40	28	31.7	− 3.7	No
41	29	33.1	− 4.1	No
42	28	33.6	− 5.6	No
43	28	33.6	− 5.6	No
44	28	33.9	− 5.9	No
45	26	32	− 6	No
46	26	34.2	− 8.2	No
		Mean difference ± SD: 2.65 ± 6.4 yrs		

Paternal age effect (PAE) is presented as higher than two years above concurrent average age according to Goriely et al. [42], which correspond to 20 of 46 fathers of registry patients

**Fig. 1:**
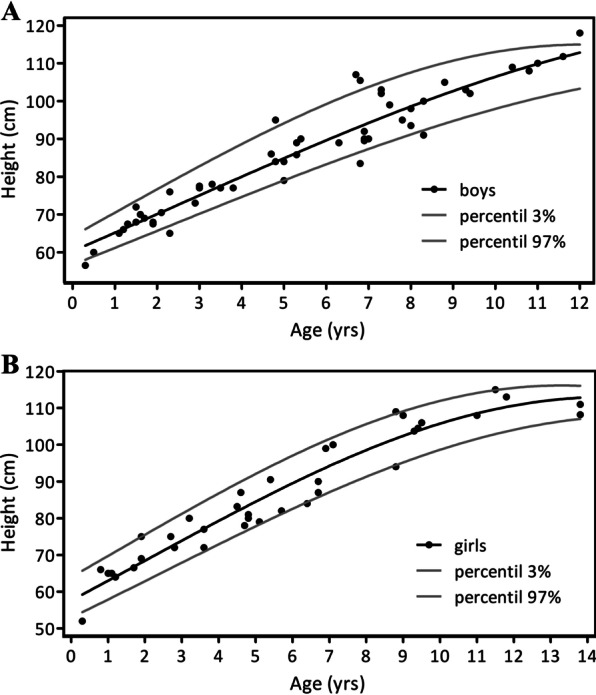
Growth charts of ACH patients. Body length measurements for girls (**A**) and boys (**B**) in centimeters at given age (years). All measurements were below the 3^rd^ percentile of regional growth curves. 97th and 3rd height percentiles are calculated (grey lines)

Stature height, body weight, and occipital-frontal circumference measurements were collected totaling 89 individual measurements (52 for 29 boys, 37 for 22 girls). Figure 1 shows stature height values for girls and boys. Repeated height measurements were obtained for 20 patients, enabling calculation of the annualized growth velocity (AGV) in 12 boys and 8 girls (Table 2). Height measurement differences were calculated as interval growth value in centimeters per year and described with annual frequency. On an individual level, AGVs were in the lowest 10% percentile of available AGV growth charts [43].

**Table 2:** Annualized growth velocity in ACH patients

Boys			Girls		
Patient No	AGV	Age measured	Patient No	AGV	Age measured
	(cm/yr)	(yrs)		(cm/yr)	(yrs)
1	8.7	1–2	1	7.3	1–3
2	7.3	0–2	2	5.5	5–7
3	7.1	2–3	3	5.2	7–9
4	6.3	5–7	4	4.7	3–5
5	6.0	3–5	5	4.5	3–5
6	5.7	10–12	6	4.2	7–9
7	4.2	8–9	7	2.2	9–11
8	3.9	1–8	8	2.0	2–6
9	3.7	2–4			
10	2.5	5–7			
11	2.2	8–9			
12	1.7	5–8			

Annualized growth velocity (AGV) calculated for 20 cases (12 boys, 8 girls). Height measurement difference is calculated as interval growth value in centimeters per year

**Fig. 2:**
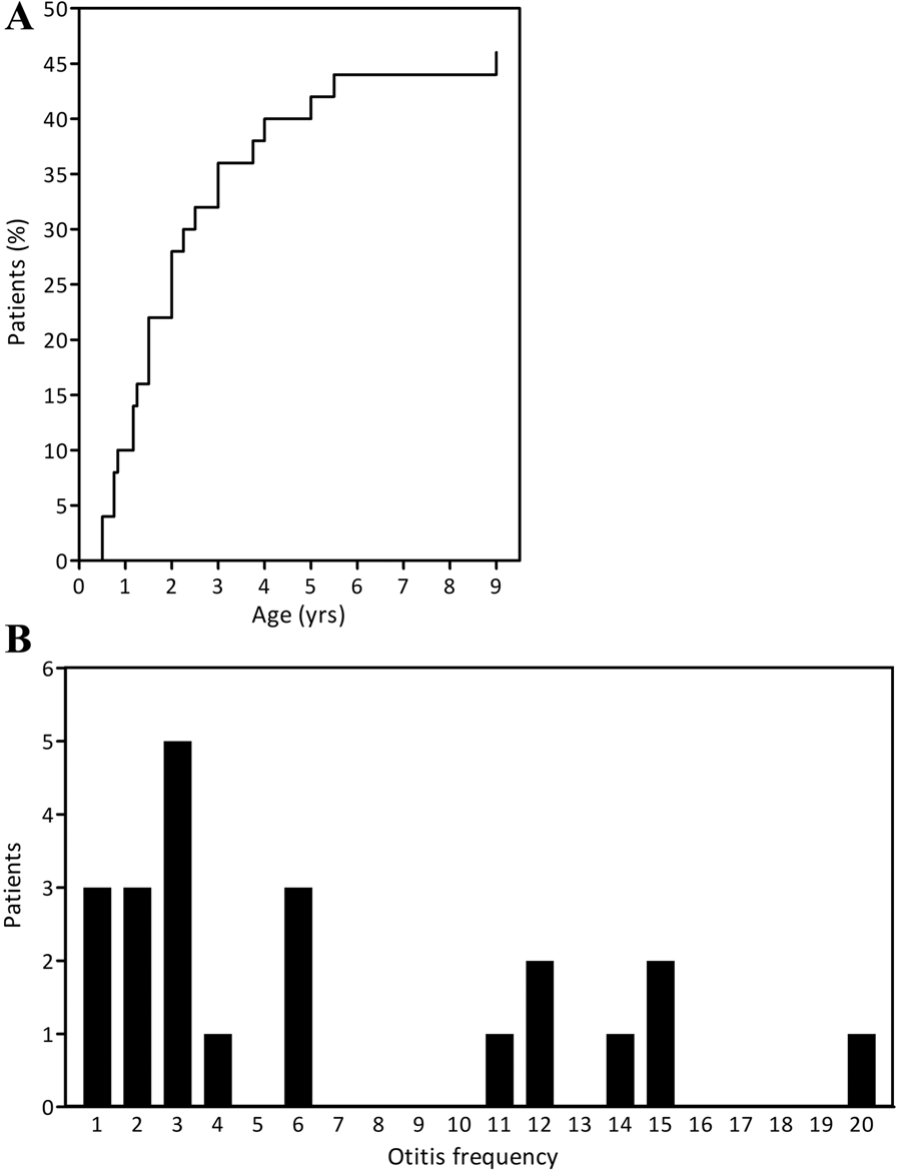
Otitis in ACH patients. Acute otitis media (AOM) affected 24 (47.1%, n = 51) patients, and was diagnosed in the majority of affected individuals in their first 2 years of life (**A**). Almost all ACH children experienced more than one episode of AOM (20/24; 83.3%), 66.7% were having more than two AOM, and 45.8%. had more than three episodes. Recorded were patients presenting 15 and 20 AOM episodes (**B**)

Among the most frequent comorbidities (Table 3) was acute otitis media (AOM), which affected 24 (47.1%) patients with ~ 50% of AOM diagnoses in the first two years of life (Fig. 2A). The first AOM occurred at 1.5 ± 0.7 years, which is earlier when compared with a large non-ACH AOM cohort showing an average age of first episode at 2.58 years [44]. 83.3% of ACH children experienced more than one AOM (Fig. 2B) in contrast to the general population where recurrent AOM is reported only in about 20.3% of children [44, 45]. These findings correspond with high incidence of chronic otitis media in ACH [45]. Moreover, 66.7% of ACH patients vs. 5.5% of the general population [48] had more than two AOMs, and 45.8% vs. 1% had more than three AOMs. Two ACH patients suffered from 15 and 20 AOMs.

**Table 3:** Comorbidities in ACH patients

	Boys (%)	Girls (%)
	n = 29	n = 22
Hypertension	0	0
Renal insufficiency	0	0
Arthritis	0	0
Osteoporosis	0	0
Back pain	13.8	18.2
Tibial malformation, bowing	69.0	81.8
Gibbus	10.3	13.6
Kyphosis	55.2	59.1
Stenosis	10.3	27.3
Radiculopathy	0	0
Spinal Surgery	3.4	13.6
Orthopedic care	93.1	100.0
Neurology care	62.1	54.5
Hydrocephalus	27.6	18.2
Snoring	44.8	40.9
Apnoe reported by parents	6.9	22.7
Apnoe diagnosed	10.3	9.1

The absence of comorbidities such as hypertension or renal failure, which is crucial for C-natriuretic peptide-based therapies for ACH [15, 46–48], was not observed in the registry cohort. No arthritis or osteoporosis was recorded. Orthopedic care was described in 49 of 51 patients (96.1%) mostly for tibial malformation, tibial bowing, gibbus, and kyphosis. Spinal stenosis and hydrocephalus were reported in 30 patients (58.8%) (Table 3) and required neurosurgical treatment in four cases. All 30 patients receive continuous neurological care. In 22 (43.1%) patients the parents reported sleep rhonchopathy (snoring) and five patients (9.8%) underwent a sleep study and had confirmed sleep apnea. One patient underwent surgical prolongation of tibial bones during their follow-up. One patient received growth hormone (somatotropin study; Novonordisk, NCT01516229). The evaluation of the quality of life was not carried-out, however, it is of substantial interest [49] and will be collected by the registry in near future.

## Conclusions

In summary, the ReACH registry collected clinical information from 51 pediatric ACH patients during its six years of existence, corresponding to ~ 60% of ACH patients aged 3 months to 14 years that were anticipated to live in the Czechia and Slovak Republic. The registry enables the first assessment of the current level of ACH diagnostics and care in both countries.

## Methods

The patient data were collected under the Masaryk University and the Office for Personal Data Protection of Czech Republic protocol no. 45/2015, approved by the institutional ethical committee on December 16th, 2015. Patients were approached by genetic counselors, general pediatrics practitioners, or through patient organizations (ospalecek.cz, palcekovia.sk). For contact, a basic online form was prepared and presented at the registry website (http://www.achondroplasia-registry.cz). Patient inclusion criteria were the following: (1) completed informed consent form, (2) clinically diagnosed skeletal dysplasia verified by genetics or orthopedic evaluation, and (3) sufficient data acquisition. For patient evaluation, a complex set of questions and check box answers was prepared as a case report form (CRF; Additional file 1) including necessary epidemiological data but also exclusion criteria for possible clinical studies. Online data collection was based on a TrialDB system (Yale University, Connecticut, USA), where a unique ID is generated for each patient, data transfer is encrypted, and the system is designed to prevent any unauthorized use. Data are stored on the central server at Masaryk University in Brno in the Oracle 9i database. During data recording, a number of pediatric practices preferred the paper version of CRF (Additional file 1), which were exchanged through postal service.

## Limitations

As the patient registration is voluntary the registry cohort does not reflect all ACH patients living in the Czechia and Slovak Republic. Several existing ACH families, known to the patient organizations, did not accept invitation to the registry or provide sufficient data and were not included in the study. These families also included familial ACH cases; no familial ACH is presented in the registry. The unequal distribution of care is obvious and results in underdiagnosed comorbidities such as sleep apnea. Leading specialization in ACH care appears to be orthopedic in Czechia [50], and endocrinology in the Slovak Republic. Precise estimation of paternal age is another limitation, as it is available only for one of the 14 existing regions in Czechia.

## Supplementary Information

**Additional file 1**. Case Report Form (CRF) used for reporting the ACH patient data by collaborating pediatricians.

## Data Availability

Please contact the author for data requests.
