# Supplementary material for: A registry of achondroplasia: a 6-year experience from the Czechia and Slovak Republic
Source: Orphanet J Rare Dis. 2022 Jun 16;17:229. doi: 10.1186/s13023-022-02374-x (PMC9205086; doi:10.1186/s13023-022-02374-x)
Supplement: Supplementary file 1 — Additional file 1. Case Report Form (CRF) used for reporting the ACH patient data by collaborating pediatricians. [file 13023_2022_2374_MOESM1_ESM.pdf]

Patient name | *Year of patient birth*

ReACH registry

PHYSICIAN CONTACTS

Name

Address (Center)

Phone

E-mail

PATIENT CONTACTS (LEGAL REPRESENTATIVE)

Name

Address (City)

Phone

E-mail

Follow-up examination

Date of examination

DIAGNOSIS

☐ Achondroplasia confirmed by molecular FGFR3 analysis (please fill in form Molecular genetic data)

☐ Hypochondroplasia

☐ Pseudoachondroplasia

☐ Skeletal dysplasia without genetic confirmation

☐ Other skeletal dysplasia – specify

Was the wrist X-Ray used to determine bone age? ☐ YES ☐ NO

If YES, were epiphysis closed? ☐ YES ☐ NO

CLINICAL DATA

Weight (kg)

Height (cm)

Head circumference (cm)

Systolic blood pressure (mmHg)

Diastolic blood pressure (mmHg)

Heartbeat frequency (beat per minute)

Specialized ambulant care

(Check the specialist checkbox if you have visited any of these specialists at least once since birth until this follow-up)

☐ General pediatrics ☐ Orthopedics ☐ Cardiology ☐ Otorhinolaryngology ☐ Neurology

☐ Ophthalmology ☐ Rehabilitation ☐ Endocrinology ☐ Anthropometry

☐ Other specialized care – specify

Patient name *Year of patient birth*

ORTHOPEDICS

Motor functions – choose current status

☐ Non-walking child ☐ Reduced ability to walk with support

☐ Normal walking ☐ Wheelchair bound

☐ Reduced ability to walk without support

Independent walking start (months) [ ]

Orthopedic diagnosis (established since birth until this follow-up)

Arthritis	☐ YES	☐ NO		Tibial bowing, lateral tibial torsion	☐ YES	☐ NO
Osteoporosis	☐ YES	☐ NO		Thoraco-lumbar kyphosis	☐ YES	☐ NO
Back pain	☐ YES	☐ NO		Spinal canal narrowing	☐ YES	☐ NO
Lower extremity radiculopathy	☐ YES	☐ NO		Hypotonia	☐ YES	☐ NO
Tibial malformity	☐ none	☐ mild	☐ severe	Presence of gibbus	☐ YES	☐ NO

Other motor limitations and abnormalities – specify [ ]

Ortopedic therapy (since birth until this follow-up)

Ortopedic surgery ☐ YES ☐ NO

If YES, choose the surgery type

- Cervical spine surgery (spinal cord oppression) ☐ YES ☐ NO
- Lumbar spine surgery (endplate overload) ☐ YES ☐ NO
- Long bone prolongation ☐ YES ☐ NO
- Other ortopedic surgery – specify [ ]

Other ortopedic therapy – specify [ ]

Supervising clinical center / specialist [ ]

HYPERTENSION AND SLEEP APNOEA

Cardiology examination

Hypertension ☐ YES ☐ NO

Current antihypertensive medication ☐ YES ☐ NO

If YES, specify the medication [ ]

Start of medication use (month and year) [ ]

Supervising clinical center / specialist [ ]

Current cardiac medication ☐ YES ☐ NO

If YES, specify the medication [ ]

Cardiac function by ultrasound (LVEF in %) [ ]

Sleep Apnoea

Fatigue during the day ☐ YES ☐ NO

Subjectively suboptimal sleep ☐ YES ☐ NO

Snoring ☐ YES ☐ NO

History of sleep apnoea ☐ YES ☐ NO

Sleep study done ☐ YES ☐ NO

Date of sleep study

Diagnosed sleep apnoea ☐ YES ☐ NO

Sleep apnoea treatment ☐ YES ☐ NO

Respiratory insufficiency ☐ none ☐ mild or occasional ☐ severe (ventilation support)

Breathing parameter FEV1 (l) (breathing parameters are available for patients older than 6 years)

Breathing parameter FVC (l)

OTORHINOLARYNGOLOGY

At least once otitis media since birth until this follow-up ☐ YES ☐ NO

If YES, at least one of the following options is required

Date of the first otitis media

If the date is unknown, write age in months (estimate)

No. of otitis media relapses (total)

NEUROLOGY

Neurological status ☐ Normal according to age ☐ Delayed

Hydrocephalus ☐ YES ☐ NO

If YES, was the surgery performed in order to release brain overpressure? ☐ YES ☐ NO

Transcranial ultrasound examination ☐ YES ☐ NO

OPHTHALMOLOGY

Specify diagnosis and treatment

REHABILITATION

Rehabilitation techniques applied

Posture rehabilitation ☐ YES ☐ NO

Contracture prevention ☐ YES ☐ NO

Other – specify technique and frequency

Patient name *Year of patient birth*

ENDOCRINOLOGY

Hormonal therapy (e.g. growth hormone) ☐ YES ☐ NO

If YES, specify

Other endocrinology follow-up (e.g. thyroid disease monitoring) ☐ YES ☐ NO

If YES, specify

Supervising clinical center / specialist

BIOCHEMISTRY

Biochemical results ☐ YES ☐ NO

Date of blood sample collection

Na (mmol/l)		Creatinine (mmol/l)	
K (mmol/l)		Erythrocytes (x 10^12/l)	
Cl (mmol/l)		Leukocytes (x 10^9/l)	
Urea (mmol/l)		Thrombocytes (x 10^9/l)	

Other results (e.g. NT-proBNP level, B- and C-type natriuretic peptide) – write date of blood sample collection and values with units

OTHER REGISTRIES

Signed up for alternative registries ☐ YES ☐ NO

If YES, specify other registry

CLINICAL STUDIES

Patient involved in clinical study (except this clinical ReACH registry) ☐ YES ☐ NO

If YES, specify clinical study / treatment / medication

COMMENT

Please write down all important information
(e.g. other than orthopedic surgery, tonsillectomy, serious injury with permanent consequences)
